# High-throughput super-resolution imaging chip based on miniaturized full-frequency encoded-illumination

**DOI:** 10.1038/s44172-026-00722-4

**Published:** 2026-07-21

**Authors:** Xiaoyu Yang, Haonan Zhang, Yuqi Zhang, Feihong Lin, Mingwei Tang, Tawfique Hasan, Clemens F. Kaminski, Xu Liu, Qing Yang

**Affiliations:** 1 State Key Laboratory of Extreme Photonics and Instrumentation, College of Optical Science and Engineering, Zhejiang University, Hangzhou, China; 2 ZJU-Hangzhou Global Scientific and Technological Innovation Center, Zhejiang University, Hangzhou, China; 3 Department of Chemical Engineering and Biotechnology, University of Cambridge, Cambridge, UK; 4 Cambridge Graphene Centre, University of Cambridge, Cambridge, UK

**Keywords:** Super-resolution microscopy, Total internal reflection microscopy, Imaging and sensing, Transmission light microscopy

## Abstract

Super-resolution microscopy has become an indispensable core research tool in biology, materials science and medicine, yet existing technologies face an inherent bottleneck where resolution, large field of view (FOV), label-free compatibility and system cost cannot be simultaneously optimized. A miniaturized full-frequency encoded illumination (mini-FEI) chip is presented for high-throughput super-resolution imaging using the spatial frequency shift (SFS) effect. We propose a tunable continuous SFS method for across far-field and near-field illumination modulation, which is achieved through propagating and evanescent waves. The multi-illumination modes are precisely and flexibly modulated by an encoded LED array. The light travels to the sample via a set of prisms, producing super-resolution images with high signal-to-noise ratio (SNR). Mini-FEI super-resolution imaging reaches a resolution of 333 nm ( ~ λ/4NA), while maintaining a large FOV of ~1 mm^2^. The method is validated on label-free samples including USAF Target, Star Target, onion root tip cells, and live COS7 cells, all of which could be successfully reconstructed. Through the introduction of integrated LED arrays for evanescent wave excitation, expensive laser systems can be avoided and the system significantly miniaturized. The mini-FEI super-resolution imaging chip is simple and cost-effective to fabricate, is compatible with commercial microscopes, and thus holds great potential for widespread deployment in scientific and industrial research.

## Abstract (web summary)

Xiaoyu Yang and colleagues propose a miniaturized full-frequency encoded illumination chip that enables cost-effective super-resolution imaging based on the spatial frequency shift effect. The mini-FEI system achieves 333 nm resolution ( ~ λ/4NA) while retaining a 1 mm² field of view, and yields robust reconstructions across diverse label-free samples including USAF targets, star targets, onion root tip cells and live COS7 cells.

## Introduction

Super-resolution microscopy overcomes the resolution limit imposed by optical diffraction, which hampers traditional microscopy techniques^1^. It has become an important tool for key discoveries in fields as diverse as biology, materials science and medical research. Various fluorescence-based super-resolution microscopic techniques, such as stochastic optical reconstruction microscopy (STORM)^2–9^, stimulated emission depletion (STED) microscopy^10–14^ and structured illumination microscopy (SIM)^15–19^ have been reported. However, these technologies rely on fluorescent labels, which are prone to photobleaching and causing cellular phototoxicity. Furthermore, these technologies are not suitable for many non-biological samples such as nanomaterials and integrated devices.

For these reasons, label-free super-resolution microscopy techniques have been developed, including the spatial frequency compression (SFC) method and the spatial frequency shift (SFS) method. The SFS method relies on large wave-vector illumination, shifting high spatial frequencies of the object into the passband of the traditional imaging system, thus relaying subwavelength spatial information to the far-field image sensor. The SFS method is thus capable of performing undistorted and high-speed super-resolution imaging. The SFS methods encompass both near-field and far-field illumination approaches. Near-field illumination methods include fiber tip^20^, nanowire^21^, complex-frequency resonance^22^, Bloch surface wave^23^, and so on. Far-field illumination methods include far-field synthetic aperture^24^, Fourier ptychographic microscopy (FPM)^25–32^ and so on.

FPM is a typical SFS-based super-resolution imaging technique achieved through far-field illumination modulation. Zheng et al. first proposed FPM^25^, using an LED array to illuminate the sample at different angles to improve the spatial resolution. However, the fundamental limit of SFS in FPM arises from the wave-vector used for sample illumination, which is inherently constrained by its propagation in free space. As a result, the space bandwidth product (SBP) is limited. Improvements in spatial resolution are possible if evanescent fields are used, providing larger wave-vectors to the sample. This led to the design of on-chip SFS structures, including nanowire ring^21^, OLED luminous films^33^, and wafers with tunable gratings^34^. However, some of these technologies may suffer from the problem of frequency deficiency, because the effective spatial frequency spectrum achievable contains empty regions as the SFS extends far beyond the cut-off spatial frequency (SF) of the microscope objective lens. Furthermore, these on-chip SFS super-resolution imaging technologies feature a small FOV, which is also a limiting factor in the achievable SBP. Another feature of current on-chip SFS technologies is the requirement for a laser source for illumination. This leads to complex external optical setups negating the advantages of on-chip integration, and hinders miniaturization of instruments and cost reduction^21,33,34^.

In this work, we present a miniaturized full-frequency encoded-illumination (mini-FEI) super-resolution imaging chip. A method of continuous SFS for across far-field and near-field illumination modulation (CS-FNIM) is proposed, which fills the entire *k*-vector space without leaving empty regions in the super-resolution OTF. This is achieved through the provision of omnidirectional SF components, which are created by both freely propagating and confined evanescent waves. The chip contains an encoded LED array, through which the multiple illumination modes can be precisely modulated. The proposed mini-FEI super-resolution imaging system features an SBP increased by an order of magnitude and an SNR enhanced fivefold over existing on-chip SFS imaging technologies. Furthermore, the mini-FEI super-resolution imaging makes use of integrated LEDs to excite the evanescent field for SFS super-resolution imaging. The technology is plug-and-play and obviates the need for complex and expensive external optical set-ups. The unit is portable and compatible with conventional microscopes. The unit is compact and simple to fabricate, requiring no nanofabrication facility or know-how, thus significantly reducing cost and offering opportunity for mass production. We validate the performance of this technology via imaging experiments on both fabricated nanostructures and biological samples, and highlight its promising application prospects in fields including biomedical research and materials science.

## Methods

In conventional optical imaging systems, the objective lens can only receive sample information contained in SF lower than cut-off frequency (${f}_{0}={NA}/\lambda$), where *NA* is the numerical aperture of the objective lens, and *λ* is the wavelength of illumination light. The objective lens can thus be regarded as a low-pass filter, which limits the spatial resolution of the imaging system. In the SFS method, high SF object information can be shifted into the passband of the imaging system by using illumination with a wave-vector ${k}_{s}$. In our imaging system, ${k}_{s}$ is defined as

1 $${k}_{s}=2\pi {f}_{s}=2\pi \frac{{n}_{eff}}{\lambda }$$

where ${f}_{s}$ is the corresponding SFS depth provided by different illumination modes, ${n}_{{eff}}=n \cdot \sin \theta$ is the effective refractive-index for individual illumination modes in the waveguide, *n* is the refractive-index of the waveguide materials, the *θ* is the oblique incident angle (the angle between the incident ray and the surface normal). Therefore, ${f}_{s}$ can be denoted as

2 $${f}_{s}=\frac{n \cdot \sin \theta }{\lambda }$$

When illuminating objects with free space illumination in air (n=1), the ${f}_{s}$ is limited to the range of $0\le {f}_{s}\le 1/\lambda$, which limits the spatial resolution of the SFS imaging system. When illuminating objects in the waveguide with n>1, the SFS ${f}_{s}$ can surpass 1/λ, leading to a further resolution improvement, and ${f}_{s}$ can be continuously shifted to cover all frequency regions without leaving gaps.

**Fig. 1:**
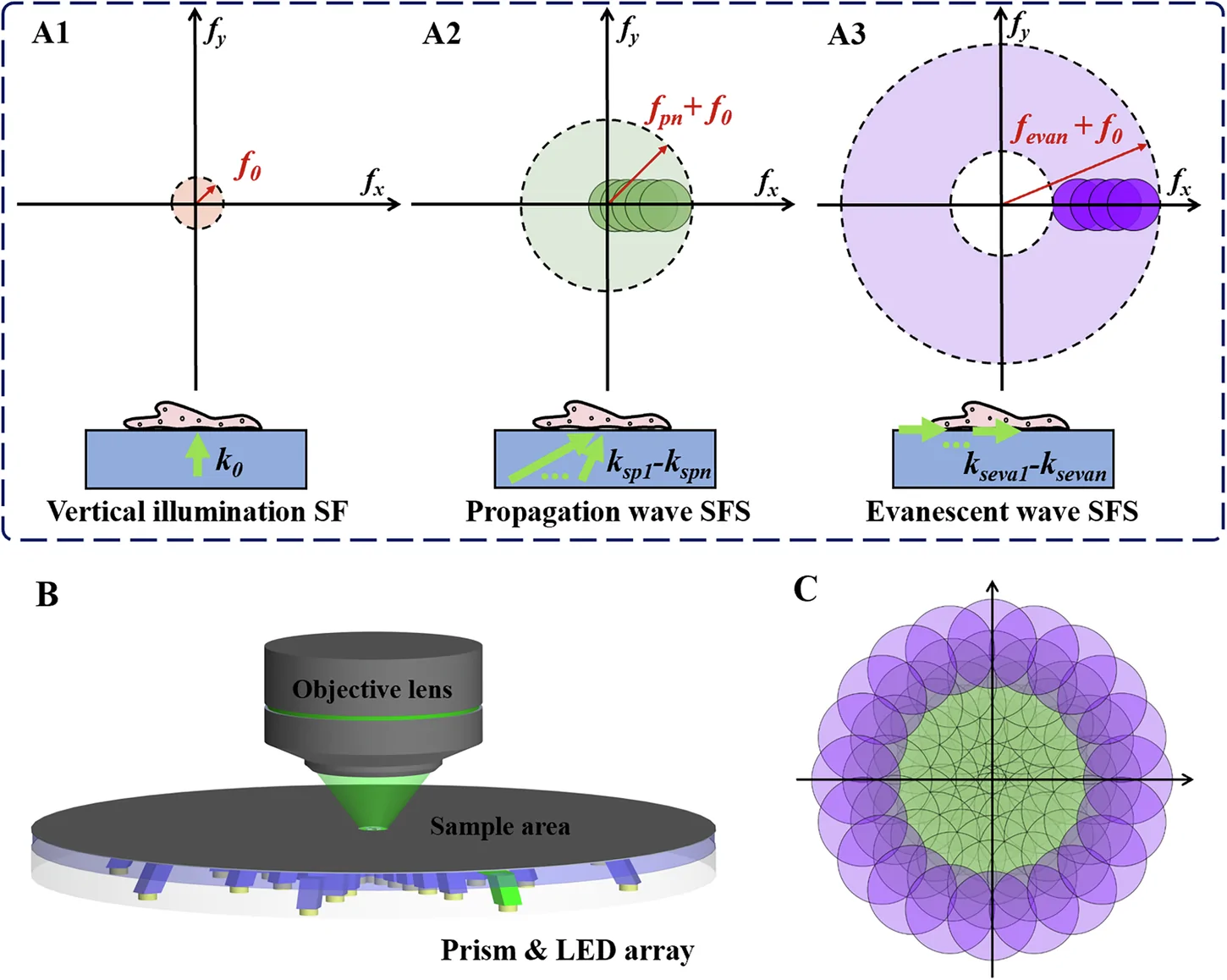
Principle of full-frequency SFS. Spatial frequencies contributed by the wave-vector under three different illumination modes: **A1** vertical illumination, **A2** oblique illumination via propagating waves, and **A3** evanescent wave illumination. **B** 3D schematic of the mini-FEI super-resolution microscopy imaging chip. **C** Corresponding spatial frequency spectrum captured using the mini-FEI imaging chip.
- When $0 < \theta < {\theta }_{c}$, the object is obliquely illuminated with wave-vector *k*_*spi*_ (*1* ≤ *i* ≤ *n*), which is provided by a propagating wave. Here $0 < {f}_{{si}} < 1/\lambda$, and the corresponding range of super-resolved SF information obtained is defined as $0 < {f}_{{sr}} < {f}_{{pn}}+{f}_{0}$, as depicted in the Fig. 1(A2).
- When $\theta \ge {\theta }_{c}$, the object is obliquely illuminated with wave-vector *k*_*sevai*_ (*1* ≤ *i* ≤ *n*), which is provided by an evanescent wave. Now ${f}_{si} \ge 1/\lambda$, and the corresponding range of SF information spans ${f}_{{eva}1}-{f}_{0} < {f}_{{sr}} < {f}_{{evan}}+{f}_{0}$, as depicted in the Fig. 1(A3). ${\theta }_{c}$ is the critical angle of total internal reflection.

Three illumination modes can be distinguished:

- When θ=0, the object is vertically illuminated with wave-vector ${k}_{0}$. The SFS is ${f}_{s}=0$, and the corresponding range of SF information obtained by SFS super-resolution imaging process is defined as $0 < {f}_{{sr}} < {f}_{0}$, as depicted in Fig. 1(A1).

We define ${\Delta f}_{s}={f}_{{s\_i}+1}-{f}_{{s\_i}}=(n \cdot \left(\sin {\theta }_{i+1}-\sin {\theta }_{i}\right))/\lambda$, *i* is the index of the shift frequency. Once ${\Delta f}_{s} < {2f}_{0}$, our method has the ability to continuously adjust the SFS so that the full SF spectrum can be covered from which a super-resolution image can be reconstructed in real space without distortion. For good performance, the overlap between shifted spectra should exceed 20% (see Note S2 and Figure S2), so that ${\Delta f}_{s} < {1.38f}_{0}$ (i.e. ${n} \cdot (\sin {\theta }_{i+1}-\sin {\theta }_{i})\le 1.38{NA}$). In contrast to FPM, which relies on free-space illumination and is thus limited to a maximum SFS of 1/*λ*^25–32^, our method employs waveguides to generate both propagating and evanescent waves. This approach not only overcomes this fundamental resolution limit but, unlike techniques using only evanescent waves^21,33^, also ensures a gapless spatial frequency spectrum, thereby avoiding information loss and reconstruction artifacts. The detailed discussion is shown in Note S1. Moreover, we discuss the effect of using incoherent LEDs for the reconstruction algorithm in Note S3, where it is shown that despite their wide spectrum, the LEDs can be modeled as single-wavelength sources in the mini-FEI algorithm.

A schematic representation of the mini-FEI super-resolution imaging chip, the corresponding spatial frequency spectrum, and the imaging system are shown in Fig. 1B, C and Fig. S5, respectively. The light source is ordinary LEDs with a center wavelength of 530 nm. A ×20/0.4NA air objective lens (Olympus, PLN20X) and ×20/0.5NA water objective lens (Nikon, MRF07220) were used. A bandpass filter with a center wavelength of 530 nm and FWHM (full width at half maximum) of 20 nm was used to improve the SNR. The imaging sensor is a commercial complementary metal oxide semiconductor (CMOS, JCOPTIX AIC-502M-USB) camera with a pixel size of 2.2 μm. The material of the waveguide is K9 glass with a refractive index of 1.52 at 530 nm. The waveguide has two parallel surfaces. The upper side serves as the imaging surface, the center of which is used as the sample holding area. To improve the SNR, all areas on the upper surface except the sample area are covered with opaque black paint. The lower surface is connected with a series of prisms arranged in concentric circles with the sample area at their center. The prism distribution is shown in Fig. S6. A full list of parameters for the mini-FEI imaging chip is shown in Table S1. LEDs are aligned vertically beneath the prism bases, supported by a 3D-printed holder. The LEDs are connected to a PCB board, which is controlled by an Arduino microcontroller. The control software for the LEDs is preloaded onto the Arduino chip, which makes the entire FEI system plug-and-play. The illumination light from different LED sources travels through prisms onto the sample area. As the distance of the prism from the center increases, the illumination angle incident on the sample increases, which in turn increases the depth of the SFS provided by propagating and evanescent waves. The oblique incident angle is set as ${\theta }_{i}$. The wave-vector produced by the oblique incident light is *k*_*i*_ = *k*_0_*n_*neff_i*_. *k*_*0*_ is the wave-vector of incident light in vacuum, which can be expressed as ${k}_{0}=2\pi /\lambda$. With a maximum incident angle of 53°, this super-resolution imaging system can realize a theoretical maximum resolution of 327 nm (~λ/4NA). The FOV of the imaging system is the smaller of the illuminated sample area (5 mm diameter) and the FOV of the objective lens (lens dependent; 1.1 mm for the stated lens). Here, an overall FOV of 1.1 mm diameter is hence achieved. These parameters yield a SBP of 3.4×10^7^, representing at least an order-of-magnitude improvement over previous work (see Table S2). In FPM, illumination at large incident angles with incoherent LEDs leads to very low signal because only a small fraction of the emitted light reaches the sample^27^. Chip-based SFS has been proposed to partially compensate for this problem through use of gratings, which use first-order diffraction to divert the incident light to the sample area at the correct angle. Modest increases in SNR have been reported in this way; however, the first-order diffraction efficiency of the gratings used was relatively low (about 20%), and gains were marginal^34^. Here we use prisms with a reflectance coating to reflect the vertical illumination from different LEDs to the sample area. The reflection efficiency is more than 90%, which increases the SNR in collected raw images. This work demonstrates for the first time the use of encoded LEDs to excite the evanescent field for on-chip SFS super-resolution imaging, replacing conventional laser-based excitation systems, which are complex and bulky to deploy. This innovation significantly miniaturizes the illumination module of super-resolution microscopes. Furthermore, the mini-FEI imaging chip eliminates the need for complex micro/nanofabrication processes, offering strong potential for low-cost industrial production.

**Fig. 2:**
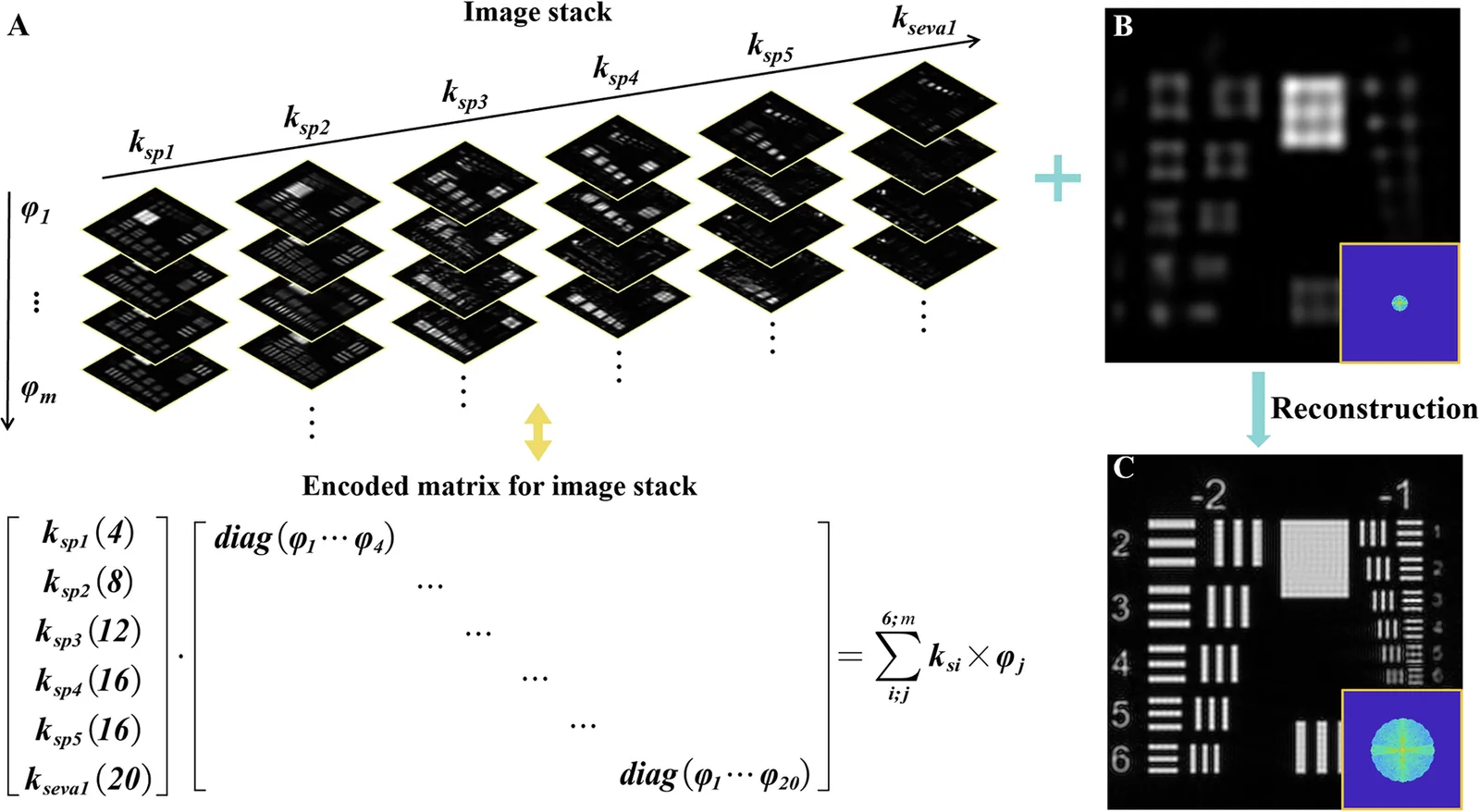
Imaging process of mini-FEI super-resolution microscopy. **A** Image stack acquired under multiple illumination  modes (top), with the corresponding encoded matrix used for reconstruction (bottom). **B** Wide-field image captured under vertical illumination. The inset displays its spatial frequency spectrum. **C** Reconstructed super-resolution image. The inset shows its enhanced spatial frequency spectrum.

During the imaging process, when a simulated wide-field image of the USAF Target vertically illuminated is obtained, it cannot distinguish any group of line pairs (Fig. 2B). This illumination corresponds to standard brightfield imaging with the associated extent of the OTF indicated in the inset of Fig. 2B. To modulate multiple illumination modes, the LEDs are controlled by the encoded matrix shown in the bottom of Fig. 2A. The LEDs are sequentially switched from the center to the outer circle, with continuously increasing SFSs including *k*_*0*_, *k*_*sp1*_, *k*_*sp2*_, *k*_*sp3*_, *k*_*sp4*_, *k*_*sp5*_, and *k*_*seva1*_. To cover the continuous SF spectrum for reconstruction, the larger SFSs (large ${\theta }_{i}$) require finer steps between different orientations *φ*_*j*_. Therefore, for each illumination circle, the LEDs are switched from *φ*_*j*_ = 0° to 360° with the steps of 90°, 45°, 30°, 22.5°, 22.5°, 18°, respectively. This means that the number of LEDs in each circle is 4, 8, 12, 16, 16, and 20, respectively (See Fig. S6). Turning on these LEDs one by one based on the encoded set, an image stack containing high SF information of the object is obtained. As shown in the top of Fig. 2A, each SFS raw image can distinguish or enhance corresponding groups of lines. Using the image stack and the wide-field image under vertical illumination, an expanded spatial frequency spectrum is iteratively reconstructed in the Fourier domain before being transformed to the spatial domain by an inverse Fourier transform, yielding a super-resolution image. All simulations were conducted in MATLAB 2020b with home-built algorithms. Additional details on the physical model simulation and mini-FEI super-resolution imaging reconstruction are provided in Note S5. A flow chart to illustrate the reconstruction algorithm is shown in Fig. S7. The theoretical resolution of the imaging system is given by ${\Delta }_{{xy}}\,=\,\lambda /({NA}+{k}_{{smax}}/{k}_{0})$, where *k*_*smax*_ is the maximal wave-vector. The period of −1-6 group of lines of the simulated USAF Target is 327 nm, which is at the theoretical resolution for the mini-FEI system. Comparing the spatial frequency spectrum of the wide-field image under vertical illumination with the reconstruction image, it can be found that the spatial frequency spectrum of the reconstruction image has expanded by 4 times, which is the key to resolving the nanostructures of objects beyond the diffraction limit. It indicates that our mini-FEI SFS super-resolution microscopy achieves 4-fold higher resolution than traditional wide-field microscopy under identical conditions.

## Result and Discussion

**Fig. 3:**
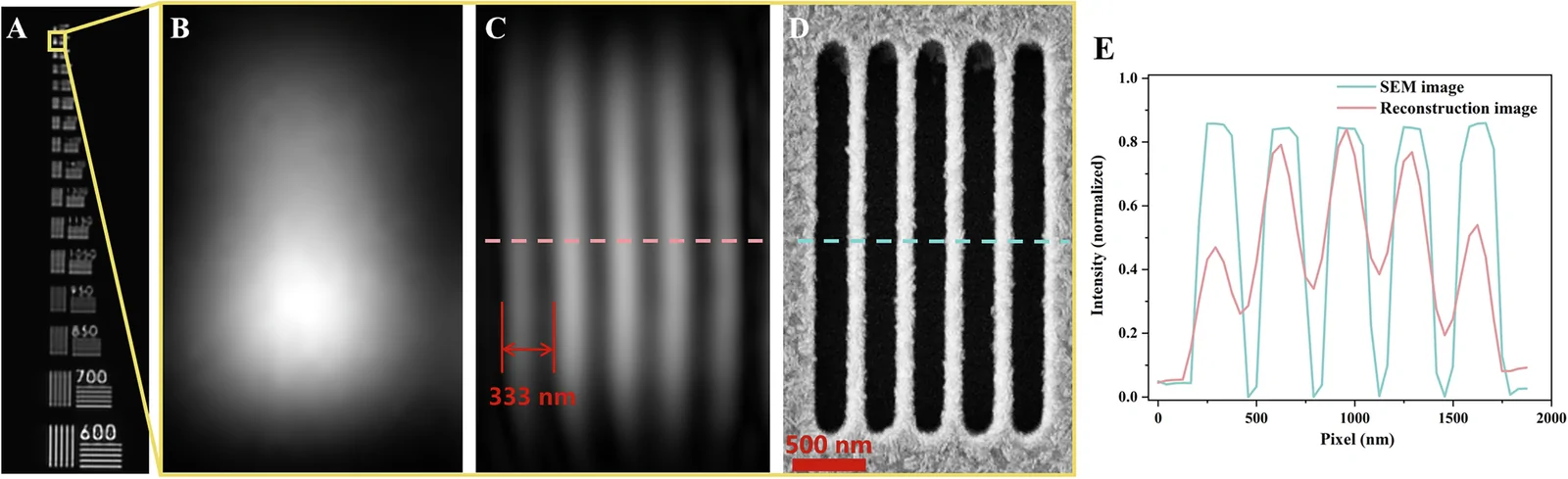
Mini-FEI super-resolution imaging of five-line pattern object. **A** Wide-field image of the five-line pattern under vertical illumination. **B** Wide-field image of the five-line pattern with 333 nm line spacing under vertical illumination. **C** The reconstruction image of five-line patterns with 333 nm resolution. **D** SEM image serving as the ground truth. **E** Normalized intensity profiles extracted along the dashed light red and light blue lines in (**C**) and (**D**), respectively, comparing the reconstructed and SEM images.

To validate our imaging method experimentally, a high-resolution USAF Target was used as the object. The object has a series of five-line patterns with different resolutions of 600 to 3300 lp/mm. The wide-field image under vertical illumination with a ×20/0.4 NA objective lens cannot resolve frequencies beyond 700 lp/mm (corresponding to a spatial period of 1429 nm), as shown in Fig. 3A. The finest structure resolvable with the mini-FEI super-resolution imaging system corresponds to a spatial period of 333 nm (the position of 3000 lp/mm), close to the theoretical limit. The wide-field image under vertical illumination at a 3000 lp/mm position is shown in Fig. 3B, which cannot be resolved at all. By using the mini-FEI, the five-line patterns with a period of 333 nm can be successfully reconstructed, as shown in Fig. 3C. A scanning electron microscopy (SEM) image of the object (Fig. 3D) was obtained as the ground truth (GT). The normalized intensity profiles of the reconstructed (light red) and SEM images (light blue) are plotted in Fig. 3E, which show good agreement with one another (Detailed information is provided in Supplementary Data 1).

**Fig. 4:**
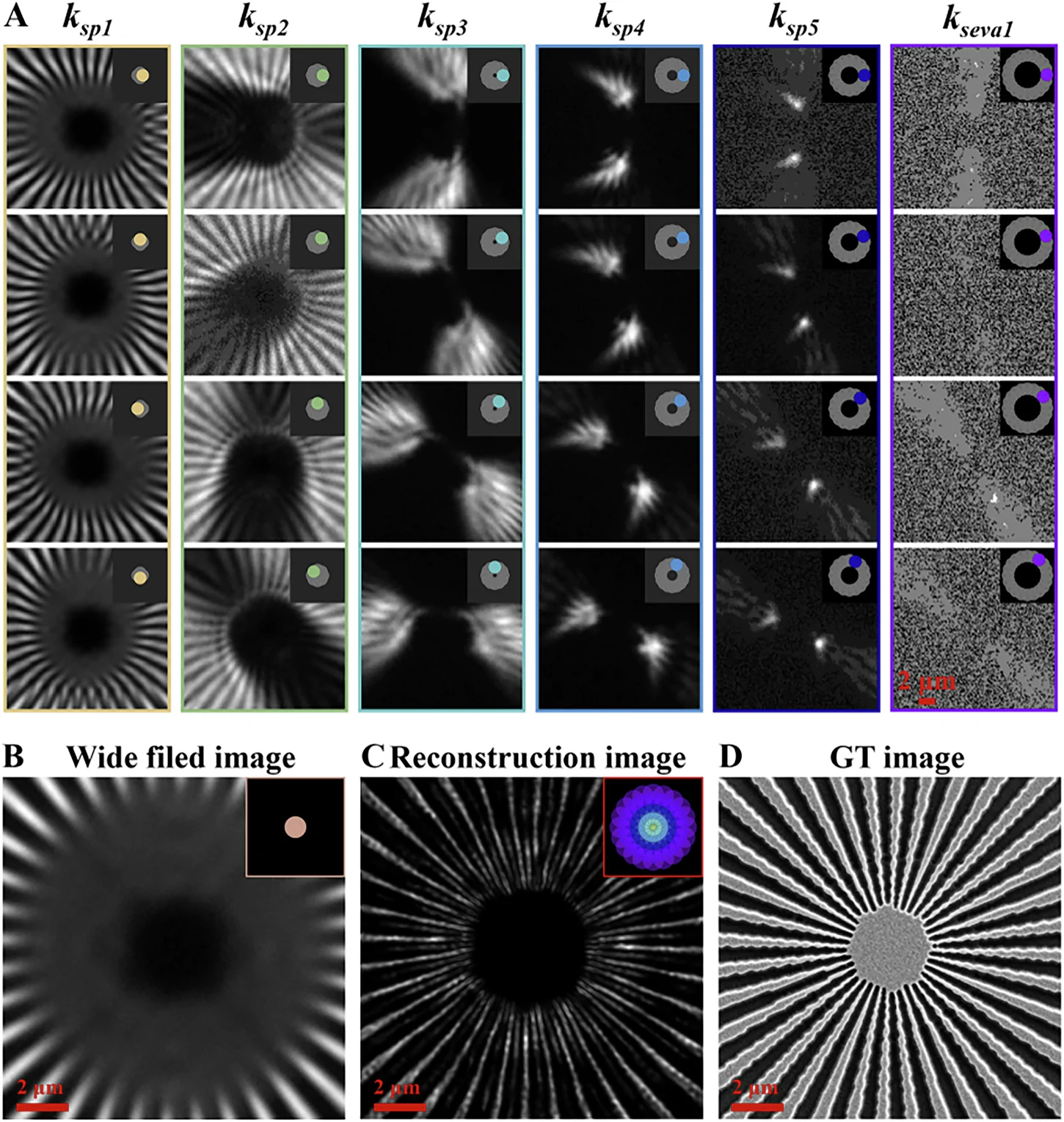
Mini-FEI super-resolution imaging of a star target object. **A** Selected SFS images captured under various illumination modes, with corresponding spatial frequency spectra shown as insets. **B** Wide-field image acquired under vertical illumination, with the associated spatial frequency spectrum illustrated in the inset. **C** Reconstructed super-resolution image, accompanied by its corresponding spatial frequency spectrum (insets). **D** Scanning electron microscope (SEM) image used as the ground truth (GT). Scale bar: 2μm.

In order to demonstrate that the method provides isotropic resolution in all directions, targets with more diverse SF information were used rather than vertical and horizontal line patterns. A Star Target was used, and raw images acquired by different illuminations are shown in Fig. 4A. Each SFS (i.e., *k*_*sp1*_, *k*_*sp2*_, *k*_*sp3*_, *k*_*sp4*_, *k*_*sp5*_, and *k*_*seva1*_) probes different parts of the SF in the sample, and different *φ*_*j*_ probe frequencies across corresponding orientations. As shown in Fig. 4B, the center of the Star Target cannot be resolved under vertical illumination (wide-field) illumination. The reconstructed image, Fig. 4C, offers much better resolution, but reconstruction artefacts are visible compared to the SEM image (Fig. 4D), which serves as the GT. The artefacts stem from the fact that the SNR in the raw images degrades for the largest SF shifts, which causes errors during reconstruction. Nevertheless, the fine structure of the Star Target is clearly resolved.

**Fig. 5:**
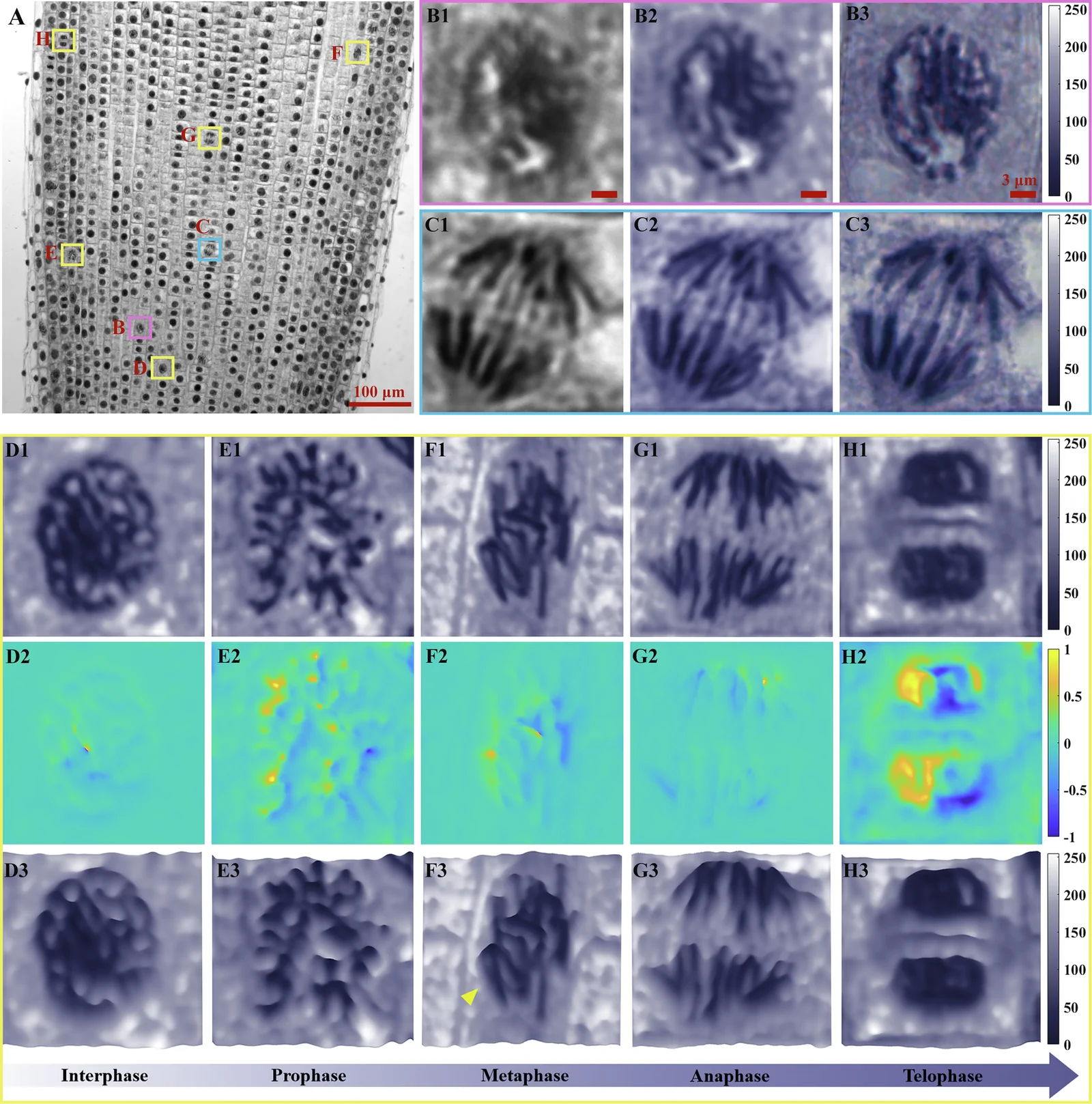
Mini-FEI Super-Resolution Imaging of Onion Root Tip Cells. **A** Wide field image acquired by a 20x/0.4NA objective under vertical illumination, showing the entire FOV of approximately 1 mm². Scale bar: 100 μm. **B1**, **C1** Wide-field images under vertical illumination of selected ROIs. **B2**, **C2** Intensity reconstruction images of the corresponding ROIs. **B3**, **C3** GT images of the ROIs, captured using a color CMOS camera with a 100×/1.49 NA oil-immersion objective. **D1**–**H1** Intensity reconstruction images of onion root tip cells at various stages of mitosis. **D2**–**H2** Corresponding phase reconstruction images of the same mitotic stages. **D3**–**H3** Three-dimensional reconstruction images showing mitotic progression. Scale bars for images (**B**) through (**H**): 3 μm.

We next applied the method to image biological samples. First, images were acquired of the root tip cells of onions. To improve the image contrast, cell sections were stained using the standard method with methylrosanilinium chloride solution. The wide field image captured by the ×20/0.4NA objective lens under vertical illumination is shown in Fig. 5A, with a corresponding FOV of ~ 1mm^2^. Two regions of interest (ROIs) within the wide field are shown in Fig. 5B1 and C1. Chromosomes present in the ROIs appear blurred. The same ROIs were also captured by a ×100/1.49 NA oil immersion objective on a color CMOS camera. These super-resolution images were used as GT for comparison (Fig. 5B3, C3). The corresponding SFS reconstructed super-resolution images obtained with the ×20/0.4NA objective lens are shown in Fig. 5B2 and C2. For better presentation and easier comparison, the SFS images were mapped onto the same color scale as the GT images. Compared with wide-field images under vertical illumination, the reconstructed images reveal finer details of the chromosomes, matching those of the GT images. There are five stages of mitosis in these cells, including interphase, prophase, metaphase, anaphase, and telophase. The intensity, phase and 3D images of these mitosis stages were successfully reconstructed, as shown in Fig. 5D1-H1, Fig. 5D2-H2 and 5D3-H3, respectively. The color bar in Fig. 5B2, B3, C2, C3, D1, E1, F1, G1, H1, D3, E3, F3, G3, H3 represents color-coded intensity. The color bar in Fig. 5D2, E2, F2, G2, H2 represents phase delay. The 3D reconstruction images indicate the thickness distribution of the sample. Compared with 2D intensity reconstruction images, the 3D reconstruction images reveal that the chromosomes are twisted across multiple planes. For instance, it can be clearly observed that two chromosomes are spatially twisted in metaphase, as marked by yellow arrow in Fig. 5F3. To verify the correction of our method, the oil objective lens of ×100/1.49 NA was used to detect the chromosomes at different focal planes within the depth of field, see Fig. S8. It denotes that 3D reconstruction images can uncover precise and comprehensive information of the sample.

**Fig. 6:**
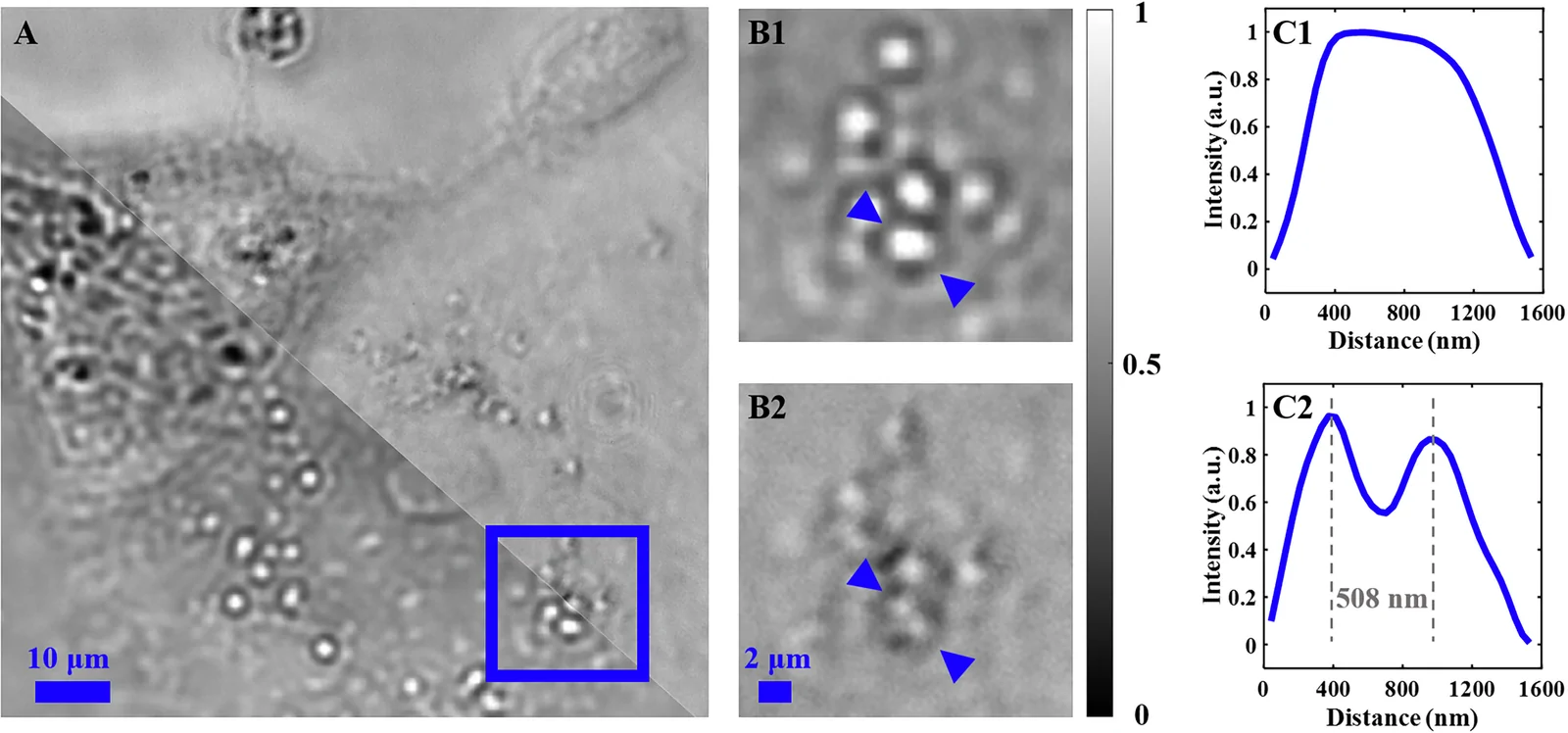
Mini-FEI super-resolution imaging of label-free live COS7 cells. A Comparison of a wide-field image (lower half) and a super-resolution reconstructed image (upper half) of the same cell. The scale bar is 10 μm. B1, B2: Magnified views of the ROI from the wide-field image (**B1**) and super-resolution image (**B2**) of the blue box in (**A**). Scale bar: 2μm. The color bar represents image intensity. (**C1**, **C2**) Normalized intensity profiles along the same line in (**B1**) and (**B2**), respectively.

Next, we used label-free live COS7 cells as samples. The COS7 cells were cultured in nutrient solution, so we used an immersion objective lens (×20/0.5NA water) to image the cells. The experimental results are shown in Fig. 6 and Fig. S9. Figure 6A shows the wide-field image in the lower half and the super-resolution reconstructed image in the upper half, clearly demonstrating the significant resolution enhancement achieved by our method. The scale bar is 10 μm. The blue box in Fig. 6A marks the ROI for detailed analysis. The ROIs of the wide-field image and the reconstructed image are magnified in Fig. 6B1 and Fig. 6B2, respectively. The scale bar is 2 μm. The color bar represents image intensity. The arrowheads highlight subcellular structures that are blurred and unresolved in the conventional wide-field image but are clearly resolved with fine details in the super-resolution image. To quantitatively verify the resolution improvement, Fig. 6C1 and 6C2 plot the normalized intensity profiles along the same line in Fig. 6B1 and 6B2, respectively. The intensity profile in Fig. 6C1 shows a single broad peak, failing to distinguish adjacent subcellular features. In contrast, the intensity profile in Fig. 6C2 resolves two distinct peaks separated by ~508 nm, enabling the visualization of fine subcellular structures in live cells without labeling (See Supplementary Data 2 for details.).

## Conclusion

In this work, a high-throughput integrated mini-FEI super-resolution imaging system is presented. The system illuminates the sample with a combination of free-space propagating and evanescent waves to permit a tunable SFS to be achieved, covering the full SF spectrum of the super-resolved object. The multiple illumination modes are precisely modulated on the chip by an encoded matrix. LED light sources are Arduino-controlled and delivered to waveguides via prisms integrated on the device. The prisms allow more than 90% of the LED emission to be delivered to the sample area, improving the SNR of the obtained images. The imaging system realizes a large SBP of 3.4 × 10^7^ with a maximal resolution of 333 nm (~λ/4NA). The mini-FEI super-resolution chip utilizes LEDs instead of lasers to excite the evanescent field, marking the first use of LEDs for this purpose. This innovation enables super-resolution imaging while significantly reducing the size of the system. The system is easy to use and compatible with standard microscope frames. No micro/nano manufacturing processes are involved in its production, significantly reducing cost and demonstrating potential for scale-up and industry use. To validate performance, we tested the mini-FEI super-resolution imaging chip with a USAF Target for maximum spatial resolution, a Star Target for resolving com plex structures, and onion root tip cells and live COS7 cells to demonstrate its potential for advanced applications such as digital pathology and neurogenetics.

## Supplementary information

Supporting Information

Description of Additional Supplementary Files

supplemental data 1

Supplemental data 2

## Data Availability

All relevant data are available from the corresponding author (Qing Yang) upon reasonable request.
