# Supplementary material for: High-throughput super-resolution imaging chip based on miniaturized full-frequency encoded-illumination
Source: Commun Eng. 2026 Jul 21;5:192. doi: 10.1038/s44172-026-00722-4 (PMC13752325; doi:10.1038/s44172-026-00722-4)
Supplement: Supplementary file 1 [file 44172_2026_722_MOESM1_ESM.pdf]

Supplementary Materials for

High-Throughput Super-Resolution Imaging Chip based on Miniaturized Full-Frequency Encoded-Illumination

Xiaoyu Yang,[1,2,3] Haonan Zhang,[1] Yuqi Zhang,[1] Feihong Lin,[1,2] Mingwei Tang,[1] Tawfique Hasan,[4] Clemens F. Kaminski,[3]* Xu Liu[1,2]* and Qing Yang[1,2]*

This PDF file includes:

Supplementary Notes 1 to 8
Figs. S1 to S9
Tables S1 to S2

Supplementary Note 1: Comparative analysis of different illumination modes.

To validate the advantages of the continuous SFS across far-field and near-field illumination modulation (CS-FNIM), we simulated four illumination modes: (1) vertical illumination, (2) oblique illumination via propagating waves (as in FPM), (3) surface illumination via evanescent waves, and (4) CS-FNIM. As shown in Fig. S1(A1), fine structures beyond the diffraction limit cannot be resolved under vertical illumination. In Fig. S1(A2), the highest-resolution features remain unresolved under oblique illumination via propagating waves (the resolution limit of FPM). Fig. S1(A3) demonstrates that under evanescent-wave illumination alone, reconstruction largely fails, and the finest structures are only faintly recovered. In contrast, as shown in Fig. S1(A4), all structures are successfully reconstructed without distortion under our CS-FNIM. Furthermore, comparing the spatial frequency spectra of propagating-wave illumination (Fig. S1(B2)) and evanescent-wave illumination (Fig. S1(B3)), the spectrum of our CS-FNIM method (Fig. S1(B4)) is not only expanded but also continuous, with no gaps between spatial frequency components. Collectively, these results demonstrate that our CS-FNIM not only achieves higher resolution than propagating-wave-based methods (such as FPM) but also avoids the image distortion typically associated with evanescent-wave-only illumination.

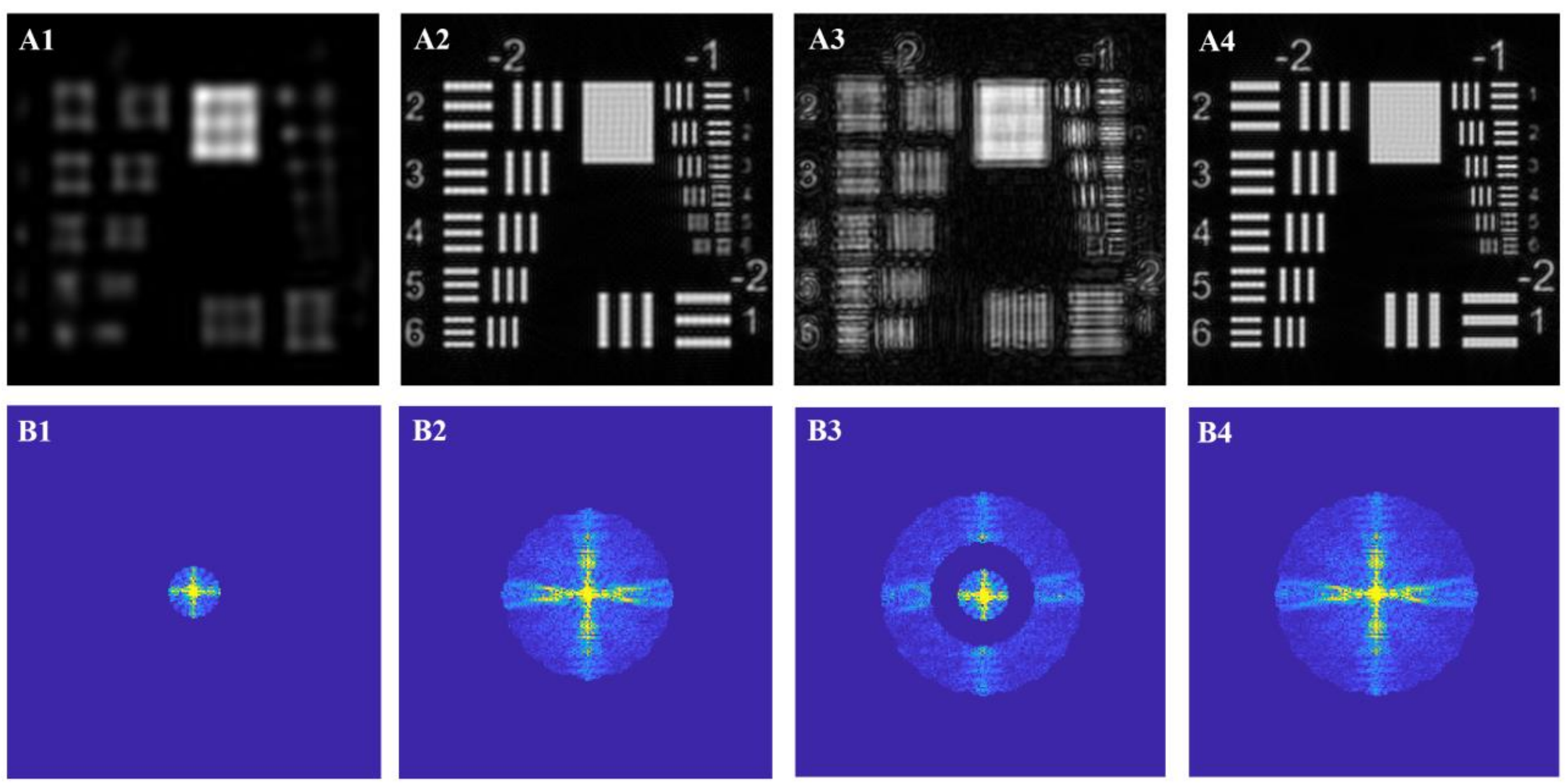

Fig. S1. Comparative analysis of different illumination modes. (A1) The wide field image and (B1) the spatial frequency spectrum under vertical illumination. (A2) The reconstruction image and (B2) the spatial frequency spectrum illuminated by propagating wave with k_{sp1}-k_{sp5}. (A3) The reconstruction image and (B3) the spatial frequency spectrum illuminated by evanescent wave with k_{seva1}. (A4) The reconstruction image and (B4) the spatial frequency spectrum illuminated by propagating wave and evanescent wave.

Supplementary Note 2: The effect of different overlap rates between SFS depths on the quality of reconstruction image.

Different overlap rates between SFS depths influence the quality of reconstruction images. Here, the root-mean-square (RMS) error between reconstruction images and ground truth image is used to value the quality of reconstruction images. A smaller RMS error indicates better quality of the reconstructed images. As plotted in Fig.S2(B), with the increase of overlap rate from 5% to 20%, the RMS error drops steeply from 0.68 to 0.31. When the overlap rate is more than 20%, the RMS error fluctuates between 0.24~0.34. As shown in Fig. S2(A), the overlap rate is below 20%, the reconstruction images are severely distorted, while the overlap rate exceeds 20%, the reconstruction images have almost no artifacts. Overall, the overlap rates between SFS depths should be set to more than 20%.

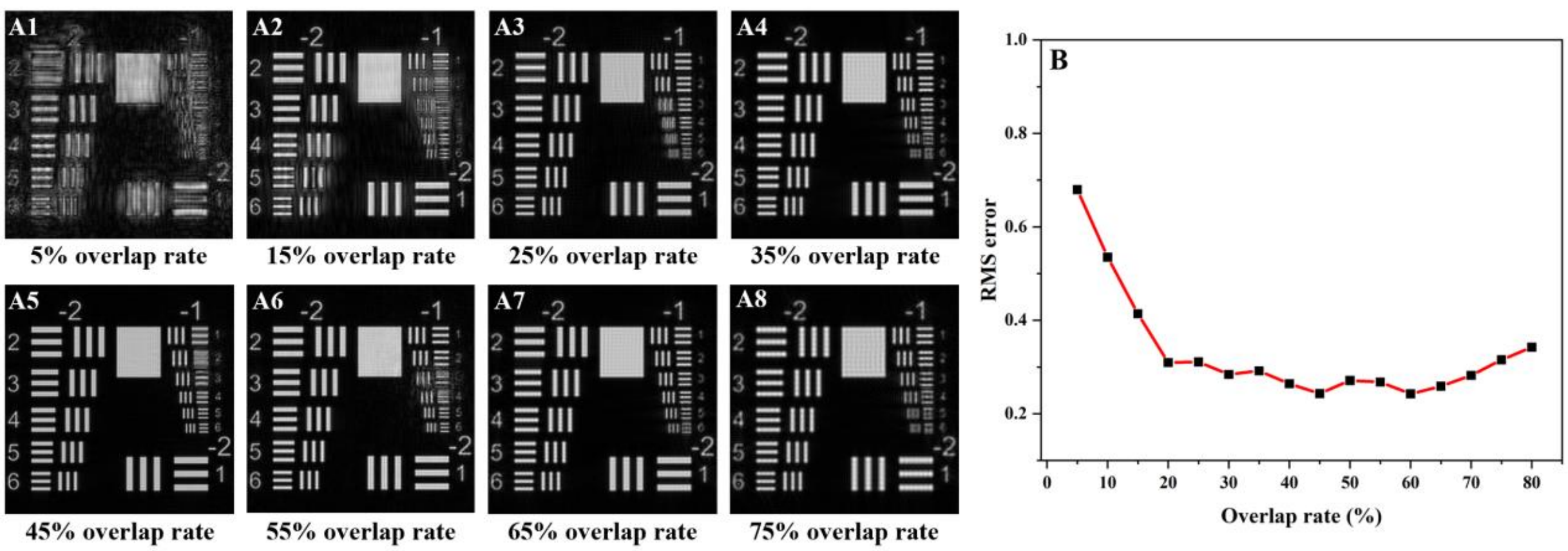

Fig. S2. The effect of different overlap rates between SFS depths on the quality of reconstruction images. (A) The reconstruction images with different overlap rates between SFS depths. (B) The RMS error of mini-FEI super-resolution imaging chip with different overlap rates between SFS depths.

Supplementary Note 3: The effects of incoherent LEDs on reconstruction images.

In the mini-FEI super-resolution imaging, the LED is regarded as the ideal coherent light source with the wavelength of 530 nm as shown in Fig. S3(B1). Its corresponding reconstruction image is shown in Fig. S3(B2), which is successfully recovered. However, the real LED used in our experiment is incoherent light source with relatively wide spectrum, whose wavelength is from 510 nm to 550 nm, centered on 530 nm, shown in Fig. S3(A1). The reconstruction image illuminated by the LEDs with wide spectrum but recovered using single central wavelength of 530 nm is shown in Fig. S3(A2), which is similar to the reconstruction image in Fig. S3(B2). It indicates that the LEDs with wide spectrum can be approximated as the LEDs with single central wavelength.

Furthermore, the central wavelength may fluctuate between 510 nm and 550 nm, influencing the quality of reconstruction images, which is displayed in Fig. S4. The LEDs used in experiment have different central wavelength between 510 nm and 550 nm, while the wavelength of 530 nm was used in the recovery process. The RMS error is firstly decreased as the wavelength is close to the 530 nm, then the RMS error is risen as the wavelength is away from the 530 nm. Notably, the RMS error is almost all below the 40% and the worst RMS error is 10% higher than RMS error in wavelength of 530 nm, which are in the acceptable range. As illustrated in the insert of Fig. S4, the finest structures of the object illuminated by LEDs with wavelengths of 510 nm and 550 nm can both be nearly successfully reconstructed, though with slight distortion, compared with those illuminated by an LED with a wavelength of 530 nm.

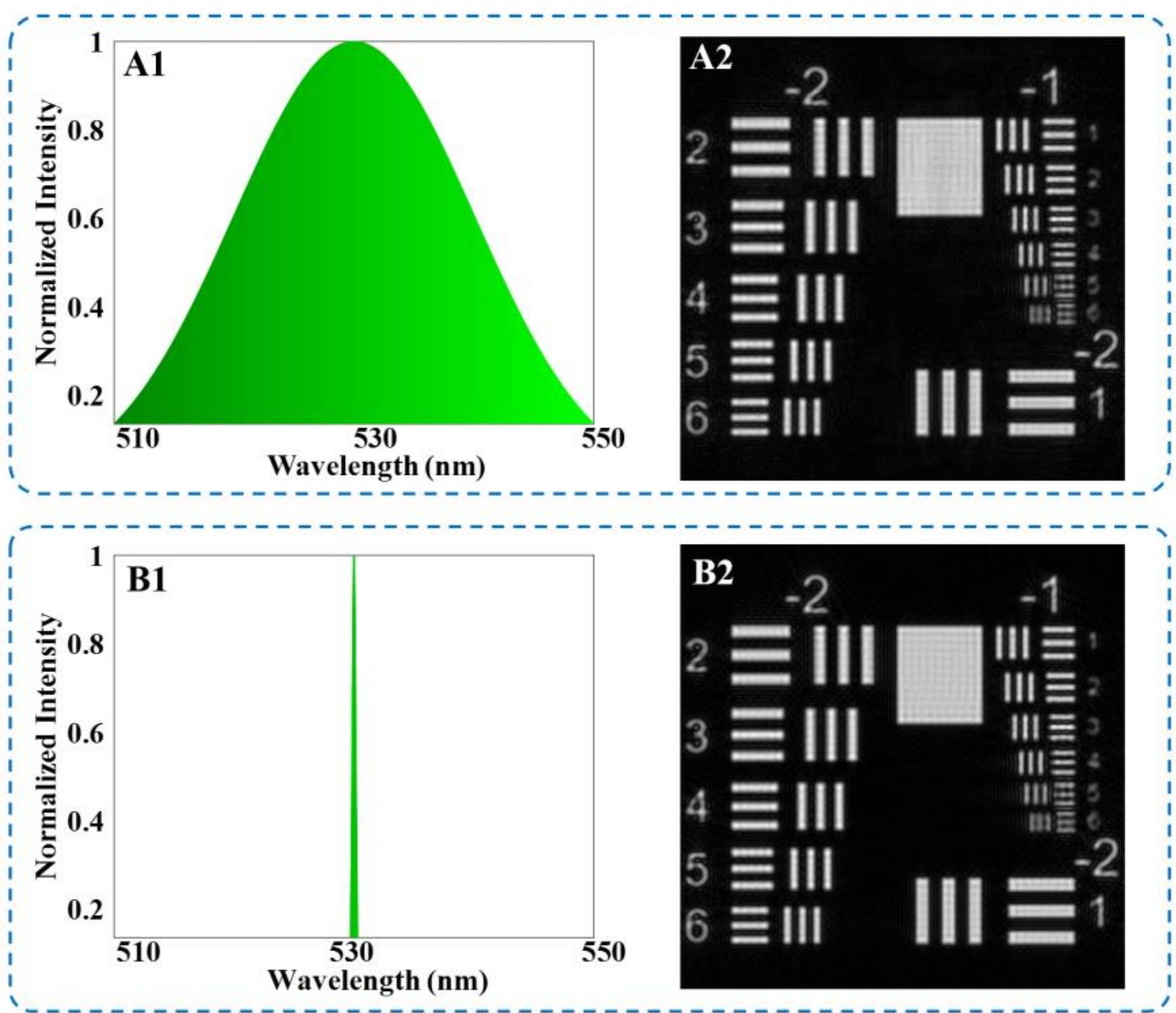

Fig. S3. The effect of incoherent LEDs on reconstruction images. (A1) The wide spectrum of LEDs; (A2) The reconstruction image illuminated by LEDs with wide spectrum. (B1) The narrow spectrum of LEDs; (B2) The reconstruction image illuminated by LEDs with narrow spectrum.

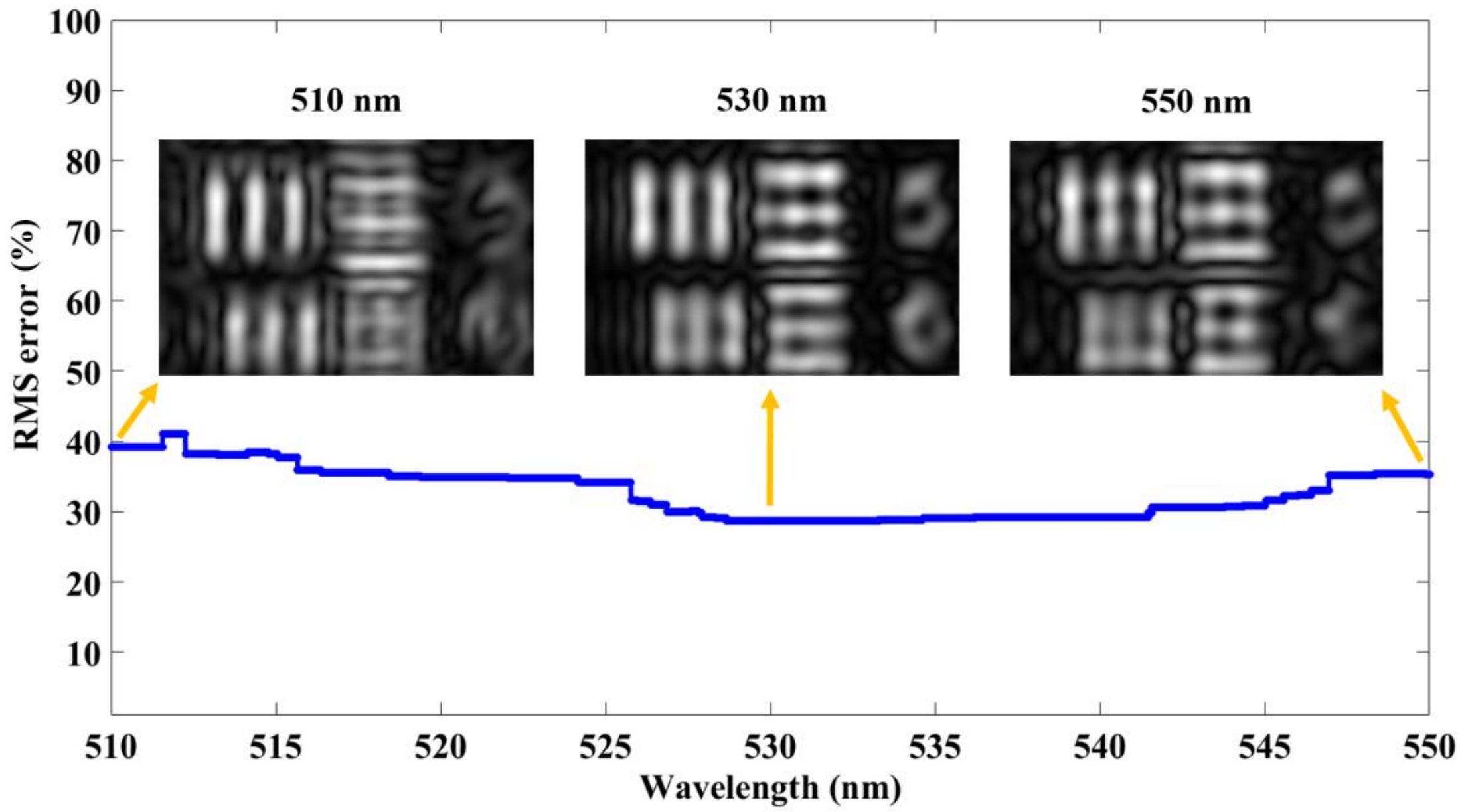

Fig. S4. The RMS error of mini-FEI super-resolution imaging chip at different illumination wavelengths.

Supplementary Note 4: The details of mini-FEI super-resolution microscopic system.

The mini-FEI super-resolution microscopic system is shown in Fig. S5(A). Among this, the physical diagram and cross-section view of the mini-FEI super-resolution imaging chip are shown in Fig. S5(B) and Fig. S5(C), respectively. The K9 glass serves as the waveguide, connected with prisms and LEDs with a 3D printed holder. Then the LEDs are connected with a PCB controlled by Arduino. The thickness of waveguide is T. The distance between the center and the prisms in each circle is L_i, with different incident angles to the sample area (θ_i = arctan (L_i/T)), which produces different k_{si}. The detailed distribution of prisms is shown in Fig. S6(A). Schematic side view of the prism is shown in Fig. S6(B). The detailed incident angle θ_i, effective refractive-index n_{eff}, overlap rate between SFS depths, the number of prisms in each circle and the distance between center to prisms in each circle L_i are shown in Table 1. The prism and waveguide are made of K9 glass, with a machining dimensional tolerance of ±0.05 mm. The surface quality is 60/40 scratch/dig, the polish particle size is 0.012 μm, and the transmittance in the visible light band is 92%.

Notably, we introduced prisms to enable straightforward on-chip integration of the light source. The normally incident light from the LED is directed onto the sample at a specific designed angle via reflective films on the prism side facets (Fig. S6(B)). Since the redirection is achieved by reflection rather than refraction, aberrations and distortions are inherently avoided. The prism position is precisely controlled using a 3D-printed holder, ensuring an error below 0.5 mm. Moreover, any residual misalignment is compensated by a position correction step incorporated into the reconstruction algorithm.

Our imaging system is compatible with all objective lenses, as its compatibility is determined by two key parameters: magnification and numerical aperture (NA). First, magnification defines the achievable FOV. The imaging chip has a FOV of 5 mm in diameter, which is larger than that of most objective lenses. Hence, from the perspective of magnification, our chip is compatible with the vast majority of objectives. Second, the overall imaging resolution depends on both the illumination NA (provided by the chip) and the detection NA (provided by the objective). Different objectives thus yield different resolutions: a higher NA leads to higher resolution. Of particular note, our chip is compatible with objectives of any NA, imposing no restriction on this parameter. Considering the trade-off between FOV and resolution, we selected a 20×/0.4 NA objective for our experiments as a balanced compromise.

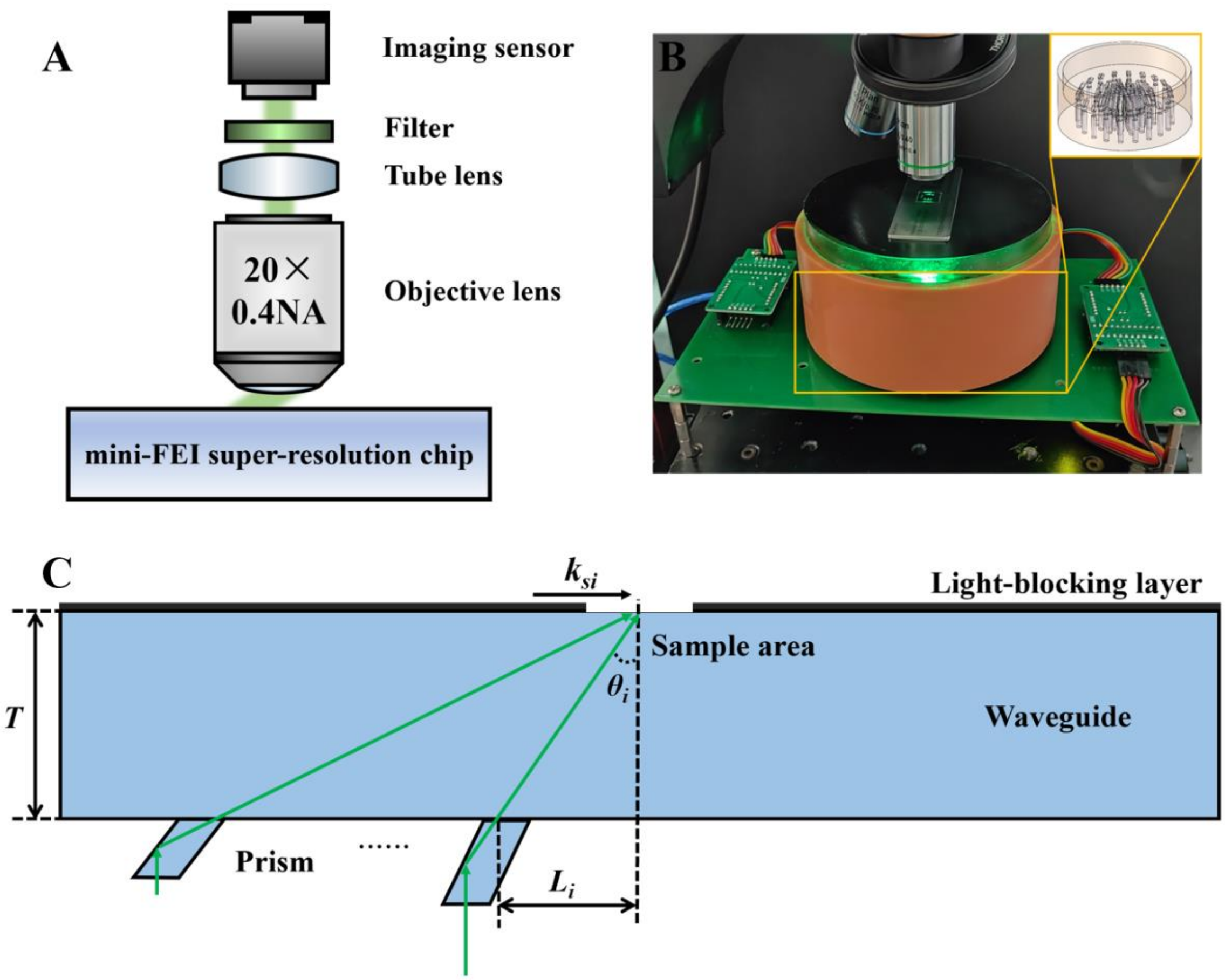

Fig. S5. The mini-FEI super-resolution imaging system. (A) The schematic of mini-FEI super-resolution microscopic system. (B) The physical diagram of mini-FEI super-resolution imaging chip. (C) The cross-section view of mini-FEI super-resolution imaging chip.

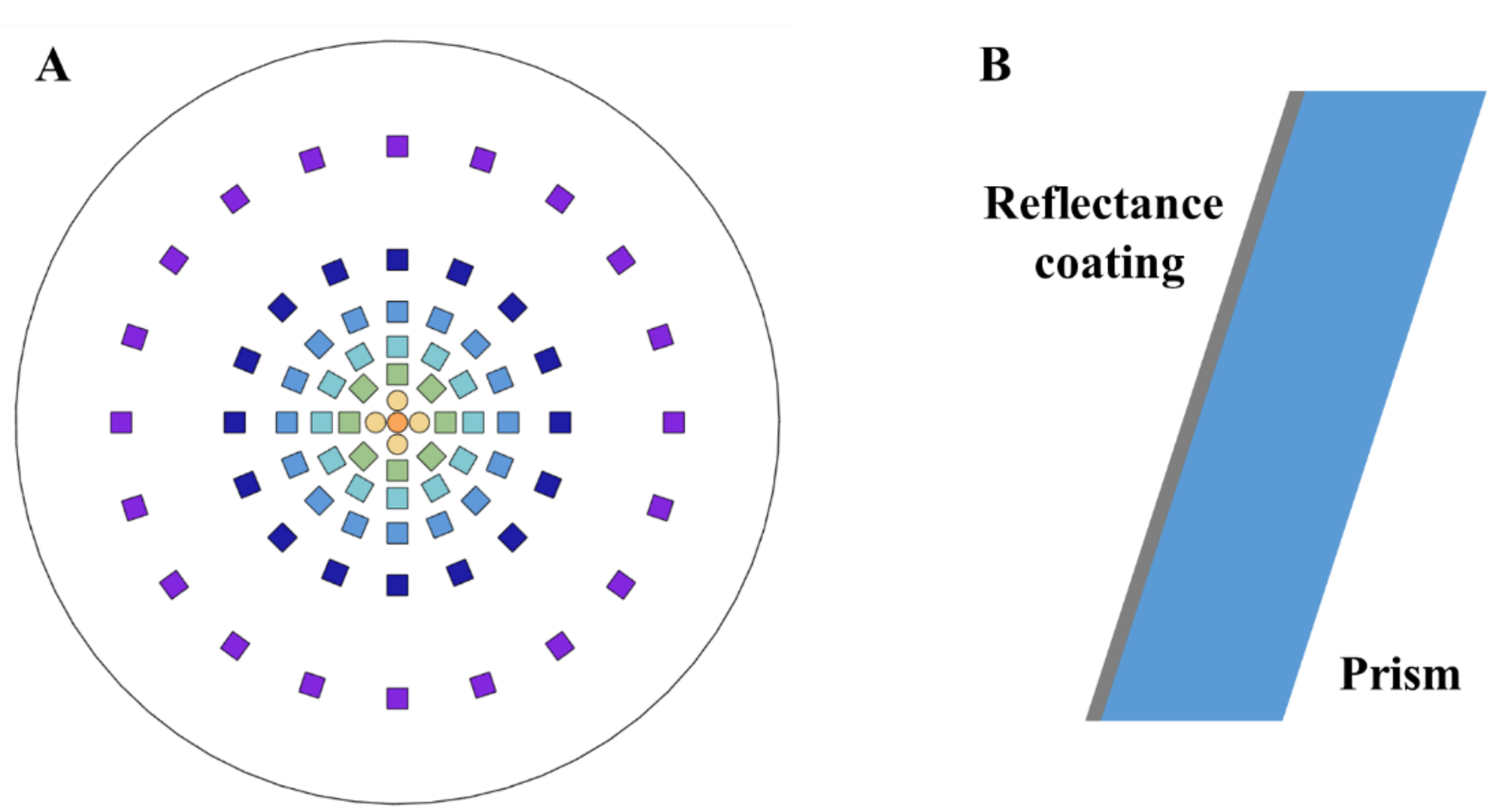

Fig. S6. (A) The schematic of prism distribution. (B) Schematic side view of the prism.

Supplementary Table 1 The parameters of mini-FEI super-resolution imaging chip

Circles	Incident angle/°	n_{eff}	Overlap rate	Numbers of the prism	*L*/mm
1	6	0.16	75%	4	3.15
2	13	0.34	72%	8	6.93
3	20	0.52	72%	12	10.92
4	28	0.72	69%	16	15.95
5	38	0.94	65%	16	23.44
6	53	1.22	56%	20	39.81

Supplementary Note 5: Physical model and recovery process of mini-FEI super-resolution imaging.

In the mini-FEI super-resolution imaging system, the transverse wave-vector of illumination field generates the SFS, which is expressed as

$$E(\vec{r}_{x,(i,\varphi)}) = \exp(ik_0 n \sin\theta_{i,\varphi})$$

Suppose the complex amplitude of objects illuminated by $E(\vec{r}_{x,(i,\varphi)})$ can be denoted as $O(\vec{r}_x)$. So that the final scattering field travelling into the objective lens can be calculated as

$$U(\vec{r}_{x,(i,\varphi)}) = O(\vec{r}_x)E(\vec{r}_{x,(i,\varphi)}) = O(\vec{r}_x)\exp(ik_0 n \sin\theta_{i,\varphi})$$

Then, the process that the complex light field $U(\vec{r}_{x,(i,\varphi)})$ is collected by objective lens is regarded as convolution process through a linear time-invariant system with transfer function $H(\vec{r}_x)$. Then the light field containing the complex information of object propagates to the plane of image sensor, which can be expressed as

$$G(\vec{r}_{x,(i,\varphi)}) = U(\vec{r}_{x,(i,\varphi)}) \otimes H(\vec{r}_x)$$

Finally, the image sensor records the intensity of the above light field, denoted as

$$I(r_{i,\varphi}) = \left|G(\vec{r}_{x,(i,\varphi)})\right|^2 = \left|U(\vec{r}_{x,(i,\varphi)}) \otimes H(\vec{r}_x)\right|^2$$

In order to analyze the SFS under different illumination modes, the Fourier transform is conducted to use as the initial input of the iteration algorithm.

$$F(f_{i,\varphi}) = \mathbf{F}\left\{I(r_{i,\varphi})\right\} = \mathbf{F}\left\{\left|O(\vec{r}_x)\exp(ik_0 n \sin\theta_{i,\varphi}) \otimes H(\vec{r}_x)\right|^2\right\}$$

The recovery process of mini-FEI super-resolution imaging is illustrated in Fig. S7. The pseudo code of recovery algorithm of the mini-FEI super-resolution imaging is shown in the following:

```
Mini-FEI Recovery Algorithm
    Input:  I(r_{i,φ}), F(f_{i,φ})
    Output: I(r), p(r)
for i = 1 to n do
    for φ = 1 to length (encoded matrix diag) do
        Δf = k_0 n sin θ_{i,φ}
        F(f) ← F(f_{i,φ} + Δf)
        I(r) ← abs(𝓕^{-1}{F(f)})
        p(r) ← angle(𝓕^{-1}{F(f)})
Output: I(r), p(r)
```

The acquisition time per raw image is 100 ms, and a total of 77 images are captured, resulting in a total acquisition time of 7,700 ms (approximately 7.7 seconds). The iterative reconstruction was performed on a computer with an Intel Core i5-13400F CPU (10 cores, 4.6 GHz) and 16 GB

RAM, using MATLAB R2020b. For a dataset of 77 128×128-pixel images, the reconstruction took about 3.5 seconds under pure CPU computation.

For an incident vacuum wavelength of 530 nm, with refractive indices n_1=1.52 and n_2=1.00, and an incident angle of 53°, the evanescent field penetration depth is calculated to be 123 nm. In this work, we adopted the weak-scattering approximation, assuming minimal perturbation of the evanescent field by the sample. Under this assumption, scattering was not explicitly modeled but treated as part of the measurable signal. However, inspired by recent advances in limited-size object microscopy (LSOM) [Chang T, et al. Super-resolution imaging of limited-size objects. Nat Photonics. 2026, doi:10.1038/s41566-026-01512-2.], we recognize that scattering effects become significant for thick or complex samples. To address this in our future work, we plan to adopt strategies from the LSOM framework—such as system transfer matrix calibration, multi-directional illumination, and spatial filtering—to mitigate scattering effects. We also aim to explore more rigorous treatments (e.g., Born series or T-matrix methods) for samples with strong scattering or high refractive index contrast in subsequent studies.

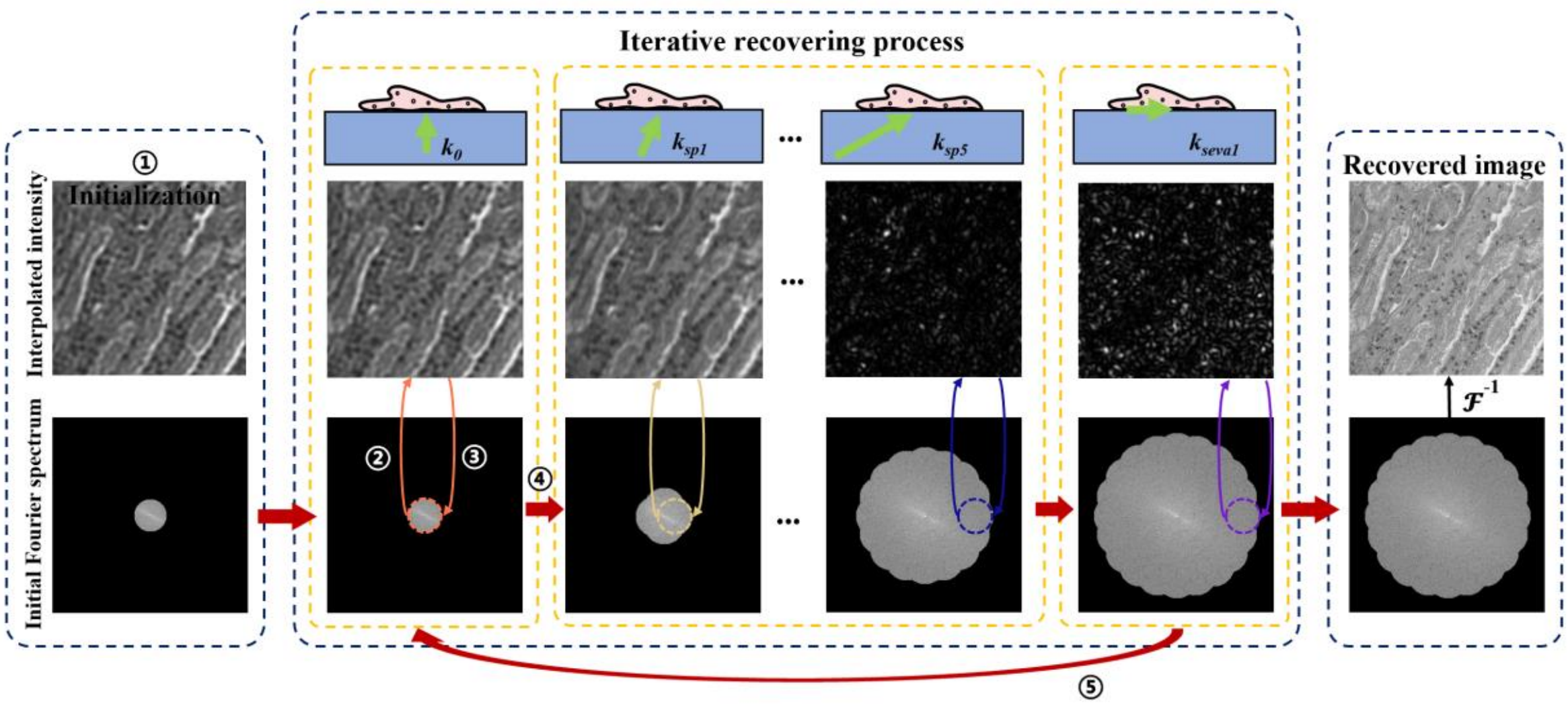

Fig. S7. The recovery algorithm flow chart of mini-FEI super-resolution imaging.

Supplementary Note 6: 3D reconstruction images containing information of different focus planes.

The 3D reconstruction image of metaphase of mitosis stage in the root tip cells of onions is shown in Fig. S8(A), observing that it contains information at different focus planes. For instance, the area marked by yellow line has different chromosomes in 3D reconstruction image, which can be verified from GT images obtained by objective lens with 1.49 NA at different focus planes, shown in Fig. S8(B1) and Fig. S8(B2). It can be seen that the information in 3D reconstruction image is nearly corresponding to the information in GT images at different focus planes.

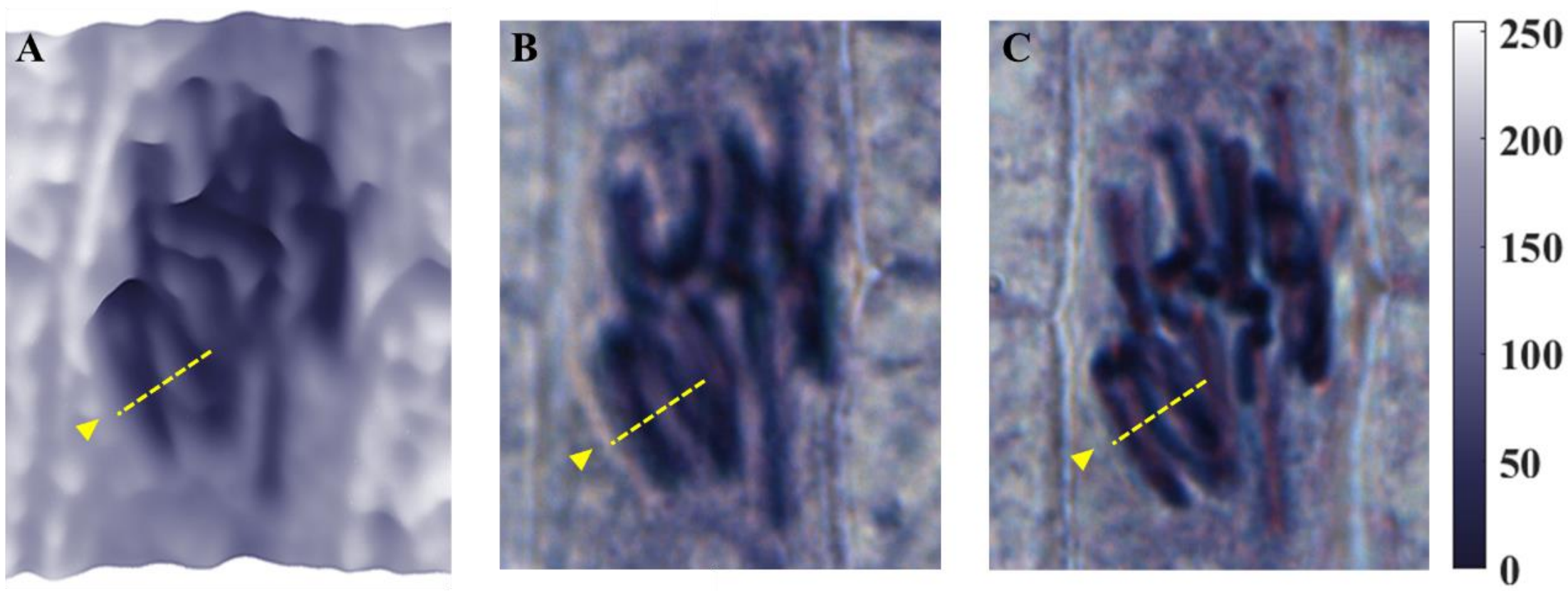

Fig. S8. 3D reconstruction images containing information of different focus planes. (A) The 3D reconstruction image of metaphase of mitosis stage in the root tip cells of onions. (B) The GT images of metaphase of mitosis stage at different focus planes.

Supplementary Note 7: Experiment results of label-free live COS7 cells.

To further demonstrate the reproducibility of our method, additional experiments were performed on live COS7 cells under the same conditions (×20/0.5 NA water immersion objective, Nikon). Representative results are presented in Fig. S9. As shown in Fig. S9(A), the wide-field image (lower half) and the super-resolution reconstruction (upper half) are compared directly, with the scale bar representing 10 µm. The ROI indicated by the blue box is enlarged in Fig. S9(B1) (wide-field) and Fig. S9(B2) (super-resolution), where the scale bar is 2 µm. The color bar indicates intensity. Subcellular features that remain indistinct in the wide-field image are clearly delineated in the super-resolution image (arrowheads). To provide quantitative evidence, the normalized intensity profiles along the same line in Fig. S9(B1) and Fig. S9(B2) are plotted in Fig. S9(C1) and Fig. S9(C2), respectively. While the wide-field profile shows only a single, unresolved peak, the super-resolution profile resolves two adjacent structures with a separation of ~586 nm. These results consistently confirm that our label-free super-resolution approach enables clear visualization of fine subcellular details in live cells, and the achieved resolution enhancement is reproducible across different cell samples.

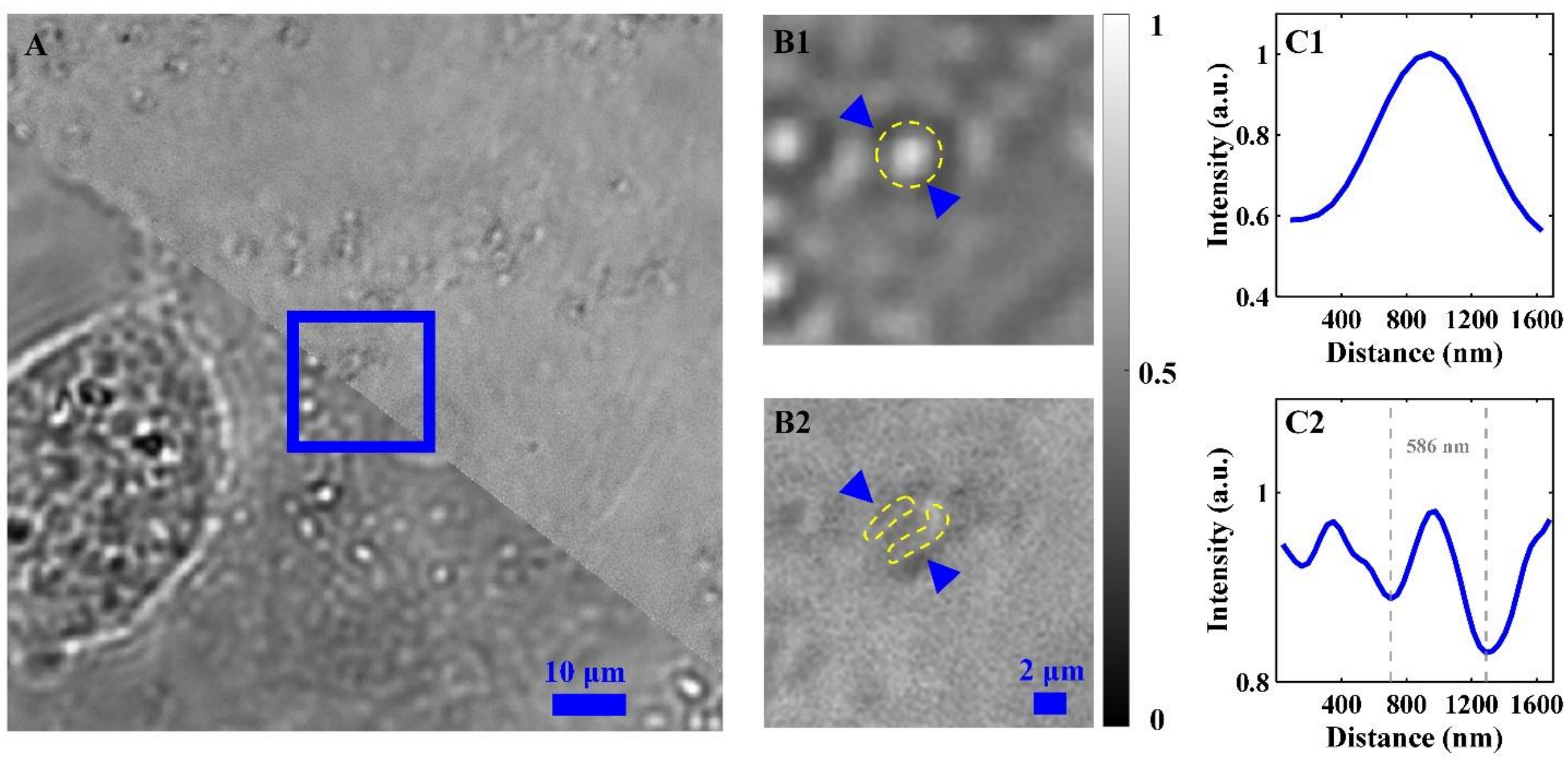

Fig. S9. **Mini-FEI Super-Resolution Imaging of label-free live COS7 cells**. (A) Comparison of wide-field image (lower half) and super-resolution reconstructed image (upper half) of the same cell. The scale bar is 10 µm. (B1, B2) Magnified views of the ROI from the wide-field image (B1) and super-resolution image (B2) of the blue box in (A). Scale bar: 2 µm. The color bar represents image intensity. (C1, C2) Normalized intensity profiles along the same line in (B1) and (B2), respectively.

Supplementary Note 8: Comparison of on-chip SFS super-resolution imaging technologies.

Based on SFS principle, the SBP of the mini-FEI super-resolution imaging can reach to 3.4×10^7, which is at least an order of magnitude higher than other on-chip SFS super-resolution imaging technologies. Moreover, comparing with other on-chip SFS super-resolution microscopy, the mini-FEI super-resolution microscopic chip is easy and cheap to fabricate without any complex and costly microfabrication process. The details are shown in Table S2.

Supplementary Table 2 Comparison of on-chip SFS super-resolution imaging technologies.

Papers	Sample type	Space bandwidth product (SBP)	Fabrication processing
Phys.Rev.Lett. (2017)	Label-free	4×10^5	Microfabrication
Adv.Func.Mater. (2019)	Label-free	3×10^6	Direct laser writing
Nat.Photon. (2020)	Labelled	3×10^4	Lithography
Nat.Commun. (2021)	Labelled	2×10^4	Lithography
Adv.Sci. (2022)	Label-free/ Labelled	1×10^5	Lithography/FIB
This work	Label-free	3.4×10^7	No microfabrication
