# Supplementary material for: High-throughput super-resolution imaging chip based on miniaturized full-frequency encoded-illumination
Source: Commun Eng. 2026 Jul 21;5:192. doi: 10.1038/s44172-026-00722-4 (PMC13752325; doi:10.1038/s44172-026-00722-4)
Supplement: Supplementary file 2 [file 44172_2026_722_MOESM2_ESM.pdf]

Description of Additional Supplementary Files

1. Supplementary Materials consist of the following items:
    ① Supplementary Notes 1–8
    ② Supplementary Figures S1–S9
    ③ Supplementary Tables S1–S2
2. Supplementary Data 1: supporting raw data for Figure 3(E).

Supplementary Data 2: supporting raw data for Figure 6(C1, C2).
